# Exploration of pulse wave analysis under reactive hyperemia and close to an arteriovenous fistula: a comparative analysis

**DOI:** 10.1186/s12872-024-04430-9

**Published:** 2025-02-04

**Authors:** Veit Busch, Sandra Müller, Joachim Streis, Timm H. Westhoff, Thomas Felderhoff, Felix S. Seibert, Stefan Reuter, Niklas Mueller

**Affiliations:** 1 Research Center for BioMedical Technology, University of Applied Sciences and Arts , Sonnenstraße 96, 44139 Dortmund, Germany; 2 Diavital, Kamen, Germany; 3 Institute for Discrete Mathematics and Geometry, Vienna, Austria; 4 Pleiger Maschinenbau GmbH & Co KG, Witten, Germany; 5 Department of Internal Medicine I, University Hospital Marien Hospital Herne, Ruhr-University Bochum, Bochum, Germany; 6 Department of Internal Medicine D, Division of General Internal Medicine, Nephrology and Rheumatology, University Hospital Münster, Münster, Germany; 7 Department of Internal Medicine III, Division of Hematology and Oncology, Hospital of the Ludwig-Maximilians-University Munich, Munich, Germany

## Abstract

**Background:**

Analyzing novel pulse wave parameters, we aimed to study specific changes in pulse waveform under high flow conditions in three collectives (i.e., healthy individuals and two collectives of patients with kidney disease and different levels of comorbidities): First, under reactive hyperemia in order to assess endothelial function. Second, close to an ateriovenous fistula in order to assess fistula function.

**Methods:**

Subjects underwent local peripheral tonometric pulse wave analysis with the SphygmoCor® device and duplex sonography to assess flow velocity (peak V_max_ and diastolic V_diast_) under physiological conditions. Corresponding measurements were then performed under reactive hyperemia and at fistula arms. The area under the curve and the mean slope between the systolic peak and the end of systole of pulse waves and duplex flow velocities were analysed as parameter differences under high flow and physiological conditions (∆A2 and ∆m2, ∆V_max_ and ∆V_diast_). In addition, the augmentation index was evaluated (only) under physiological conditions. The Wilcoxon test was used to assess parameter differences and linear correlation was performed.

**Results:**

A total of 108 subjects were evaluated (23 healthy and 85 with fistula in two distinct collectives *n* = 39/45, measurements under reactive hyperemia in 62 individuals). Significant increments in the novel pulse parameters were observed under reactive hyperemia and near a fistula and were found to correlate with corresponding changes in flow velocity (reactive hyperemia: ∆A2 and ∆m2/V_max_ r = 0.347, *p* = 0.006 and r = 0.374, *p* = 0.003; fistula: ∆A2/∆V_max_ r = 0.315, *p* = 0.003, no significant correlation for ∆m2/V_max_). Consistent with their different vascular status and endothelial function, changes in pulse wave parameters during reactive hyperemia were significantly different in patients and healthy subjects. Both high flow conditions induced similar changes in the pulse waveform and a delay of the systolic peak in all three collectives. The augmentation index was different in the three collectives and correlated with the increase of the novel parameters and the peak flow velocity under reactive hyperemia: ∆A2 r = 0.445, *p* < 0.001, ∆m2 r = 0.338, *p* = 0.007, ∆V_max_ r = 0.460.

**Conclusion:**

Detection of changes in pulse waveform under high flow conditions is potentially a new clinical application to characterize endothelial function and the functional status of ateriovenous fistulas.

## Introduction

The endothelium plays a vital role in the development of atherosclerosis and is of therapeutic and preventive interest. Pulse waveform analysis (PWA) is a useful tool to assess vascular function [1, 2], mainly in terms of vascular stiffness and central pulse wave augmentation index (AI) as a result of peripheral pulse wave reflection [3–5]. Moreover, PWA has been shown to predict cardiovascular risk in various clinical contexts [6–8].

The assessment of endothelial responsiveness, e.g. after ischemia, is an established method to test microvascular function, i.e. the ability to regulate peripheral perfusion by interaction of the endothelial intima with the blood [9, 10]. Like PWA it has been shown to be valuable in cardiovascular risk estimation and risk stratification, particularly in the preclinical and early stages of cardiovascular disease [9, 11, 12].

So far, pulse waveform-alteration under the condition of reactive hyperemia has not been analysed in depth, and studies using PWA in the context of pharmacologic modulation of endothelial function have shown little correlation [10, 13]. The effect of different endothelial functional states on the pulse waveform in previous studies relied on parameters to characterize vascular stiffness and central pulse wave augmentation [14, 15] or required additional specialized equipment with limited ability to perform full PWA [16–18].

We have recently demonstrated that PWA of upper arm arteries can be useful in the monitoring of arteriovenous fistulas (AVFs) used for hemodialysis [19–21]. A key feature of AVFs’ impact on the pulse waveform is the high arterial flow difference between the fistula and non-fistula arm, and we have shown that the magnitude of the inter-arm difference in peak flow velocity correlates with alterations of the pulse waveform [19].

Because blood flow is increased in both conditions, reactive hyperemia and close to AVFs, we wanted to use newly derived parameters of digitized PWs to compare changes in pulse waveform induced by reactive hyperemia to assess endothelial function and by AVFs to assess the AVF function.

## Materials and methods

### Study enrollment

This post hoc analysis includes individuals older than 18 years, who were hemodynamically stable, not pregnant, not breastfeeding and not suffering from psychiatric illness. They were part of two collectives (Kidney1 and Kidney2) of hemodynamically stable patients with AVF and a collective of young vascular healthy individuals and therefore without endothelial dysfunction (Healthy). Subjects were participants of two previous clinical studies [19, 21]. All measurements were performed by the same investigator in both studies, but the investigators and the subjects enrolled differed between the two studies. Members of the Healthy and Kidney1 collective are a subset of participants in a pilot study evaluating pulse wave analysis to assess fistula function of mainly kidney transplant recipients with a still functional AVF [19], in whom pulse wave analysis was successfully performed also under the condition of reactive hyperemia. Data from the Healthy collective have not yet been published. Members of the Kidney2 collective are participants of a study on oscillometry to detect low fistula flow in real clinical practice [21], in whom additional tonometric measurement data is available.

Subjects with a non-occluded fistula at the contralateral arm, a prosthetic arteriovenous grafts or central venous catheters, with acute infection or kidney failure were excluded. In the Kidney1 collective patients with upper arm fistula were also excluded.

### Protocol

All measurements were performed in supine position at room temperature. As an initial vascular assessment all subjects underwent pulse wave analysis at the radial artery of the non-dominant (Healthy) and the non-fistula arm (Kidney1 and Kidney2) with the SphygmoCor® device (applanation tonometry; version 8.2, AtCor Medical PTY LTD, U 11 West Ryde Corporate Centre 1059–1063 Victoria Rd West Ryde, New South Wales, 2114 Australia).

Measurements under reactive hyperemia and corresponding measurements in the physiological state were performed at the brachial artery of the non-fistula (Kidney1 collective) or the non-dominant arm (Healthy collective). In subjects with fistula (Kidney1 and Kidney2) pulse waves were assessed at the radial artery of the fistula and non-fistula arm. Additionally, the same procedure was carried out at the brachial artery of the fistula arm in the subjects of the Kidney1 cohort.

For reference and complementary purposes, corresponding duplex sonographic measurements were performed on the brachial artery at the non-dominant (Healthy) or non-fistula arm (Kidney 1) under physiological and hyperemic conditions, as well as at the fistula arm (Kidney 1 and Kidney 2).

Hyperemic measurements were performed directly after releasing an upper arm cuff inflated with supra-systolic pressure for three minutes in total.

The measurements were approved by the local ethics committee (Healthy and Kidney1: Westfälischen Wilhelms-Universität Münster’, No. 2014–360-f-S; Kidney2 Ruhr-Universität Bochum, No.15–5279). All Participants provided informed consent which was written.

### Evaluation algorithm and analysed parameters

We processed digitized curves recorded with the SphygmoCor® device by the use of MATLAB®. In order to characterize the impact of reactive hyperemia and the hemodynamic state close to an AVF on pulse waveform we calculated novel parameters, we established in earlier work [19]. Averaged waves comprising one heart cycle with a sample rate of 128/sec were processed with the MATLAB® resampling function resulting in 408 data points/heartbeat. Thereafter, the wavelength and systole duration were normalized to 800 ms and the product from the systole duration as computed by the SphygmoCor® device and the ratio of 800/original wavelength, respectively. We now focused on the area under the curve (A2) and the mean slope (m2) in the second section of pressure pulse waves, i.e. between the systolic maximum and the end of systole.

In the initial unilateral assessment at the radial artery (see Study enrollment) the augmentation index AI computed by SphygmoCor© as ratio of aortic augmentation and pulse pressure normalized to a heart rate of 75 per minute, which is only validated for measurement at the radial artery, as well as unilateral A2 and m2 were assessed as parameters.

All other parameters were calculated from a pair of two corresponding recordings (see Protocol): Under hyperemic and physiological conditions (Healthy and Kidney1) and also at the fistula arm (Kidney1, Kidney2) as a third hemodynamic state.

As suggested by Malik et al. brachial artery blood flow velocity before and immediately after ischemia was used to characterize endothelial function [22]: The systolic peak flow velocity V_max_ and the diastolic flow velocity V_diast_ were considered as standard duplex-sonographic parameters.

Corresponding parameters in the various states (hyperemic versus physiological state, hyperemic versus fistula and fistula versus physiological state) were analysed as their difference in the respective two different states, which is indicated by a prepended *∆* in the parameter denotation.

### Statistical analyses

Standard univariate statistical analyses were used for description of demographic and clinical parameters. The medians of cohorts were compared with independent-samples median test. For pairwise sub-analysis also the Kruskal–Wallis Test was applied in case homogeneity could be confirmed by the Levene test. Differences of corresponding parameters were analysed with the help of Wilcoxon signed rank test (hypothesized median of 0). After confirmation of homogeneity Mann–Whitney U Test was used for intergroup comparison. For the analysis of the correlation between PW and duplex sonographic parameters Pearson correlation was applied. Correlation coefficients *r* and associated *p*-values are given and a linear regression was performed in case of significant testing. Endothelial dysfunction, assessed by duplex sonography under hyperemic and physiological conditions, was evaluated by statistical ROC-analysis and the result is presented as specific AUC with 95% confidence interval and associated *p*-value. The cut-off value was defined as below the 85%-percentile of ∆V_max_/∆V_diast_ of the healthy subgroup. *Significance* refers to local, unadjusted two-sided *p*-value < 0.05. Statistical analyses were performed using IBM SPSS Statistics for Windows, Version 29.0, Armonk, NY, USA (IBM Corp. Released 2022).

## Results

### Study population

We included a mixed cohort ($\frac{m}{f}: 1.77$, age: 53.1 ± 20.1 years, BMI: 25.3 ± 4.46 $\frac{kg}{{m}^{2}}$), consisting of 108 individuals (23 healthy individuals, 39 in the Kidney1 collective and 46 in the Kidney2 collective, Table 1). The healthy subjects were younger than the patients of the Kidney1 and Kidney2 collective (age 25.9 ± 8.3, 55.0 ± 11.4 and 65.2 ± 17 years, respectively). A total of 35 patients in the Kidney1 collective had a functioning kidney transplant. All subjects of the Kidney2 collective and four of the Kidney1 collective were on hemodialysis. The details of comorbidities of patients are presented in Table 1. The prevalence of all comorbidities was higher in the Kidney2 than in the Kidney1 collective.

**Table 1:** Subjects characteristics

	Whole collective	Healthy	Kidney1	Kidney2
Number	108	23	39	46
Gender (f/m)	39/69 (36.1%/63.9%)	13/10 (56.5%/43.5%)	13/26 (33.3%/66.7%)	13/33 (28.3%/71.7%)
Age [years]	53.1 ± 20.1	25.9 ± 8.3	55.0 ± 11.4	65.2 ± 17.0
BMI [$\frac{kg}{{m}^{2}}$]	25.3 ± 4.46	21.9 ± 2.1	26.3 ± 4.7	26.2 ± 4.3
Heart failure (> NYHA I)	10 (9.3%)	0 (0%)	3 (7.7%)	7 (15.2%)
Coronary heart disease	25 (23.1%)	0 (0%)	10 (25.6%)	15 (32.6%)
Peripheral arterial disease	10 (9.3%)	0 (0%)	3 (7.7%)	7 (15.2%)
Arterial fibrillation	20(18.5%)	0 (0%)	6 (15.4%)	14 (30.4%)
Current arterial fibrillation	14 (13.0%)	0 (0%)	1 (2.6%)	13 (28.3%)
Hypertension	80 (74.1%)	0 (0%)	36 (92.3%)	44 (95.7%)
Diabetes	70 (64.8%)	0 (0%)	13 (33.3%)	25 (54.3%)
Chronic obstructive pulmonary disease	15 (13.9%)	0 (0%)	1 (2.6%)	14 (30.4%)
Current Smoker	10 (9.3%)	0 (0%)	2 (5.1%)	8 (17.4%)
Functioning kidney transplant (without dialysis)			35 (89.7%)	0 (0%)
Current Hemodialysis			4 (10.3%/)	46 (100%)
Dialysis vintage [month]			50.1 ± 5.08	41.8 ± 6.3
Fistula side (right/left)			7/32 (17.9%/82.1%)	16/30 (34.8%/65.2%)
Fistula-location (forearm/upper arm)			39/0 (100%/0%)	30/16 (65.2%/34.8%)

Dialysis vintage in case of kidney transplant: Duration of dialysis until transplantation

*Abbreviations*: *f* female, *m* male, *BMI* body mass index, *NYHA* grade of heart failure according to the New York Heart association classification, *Healthy* collective of vascular healthy individuals, *Kidney1* collective of patients with chronic kidney disease and fistula, *Kidney2* collective of patients with fistula on hemodialysis

### Unilateral measurements at the radial artery

The values of AI, A2, and m2, as measured unilaterally at the radial artery under the physiological condition of non-fistula arms, exhibited notable differences between all three collectives. The medians of all three parameters could be ranked across the collectives in the order Healthy, Kidney1 and Kidney2. AI was lowest in the Healthy collective, whereas A2 and m2 were highest in the Healthy collective (Table 2). Using the Kruskal–Wallis Test after confirming homogeneity by the Levene-Test, the pairwise comparison demonstrated significant differences for AI and A2 between the Healthy and Kidney1 as well as between the Healthy and Kidney2 collective, but not between the kidney 1 and kidney 2 collective. The median of m2 exhibited a statistically significant difference between the Healthy and Kidney2 collectives, as well as between the Kidney1 and Kidney2 collectives. However, no statistically significant difference was observed between the Healthy and Kidney1 collective (with the independent-samples median test, since homogeneity could not be proven for m2).

**Table 2:** Comparison of unilateral measurements at the non-fistula arm across sub-collectives

	**HealthyMedian (min/max)**	**Kidney1 Median (min/max)**	**Kidney2 Median (min/max)**	*p*
**AI** [%]	4.00 (-30/23)	15.00 (-13/33)	22.00 (0/39)	< 0.001
**A2**_**non-fist**_ [relative amplitude*s]	0.17990 (0.12995/0.238938)	0.15480 (0.06830/0.20816)	0.12887 (0.06050/0.22675)	< 0.001
**m2**_**non-fist**_ [relative amplitude/s]	-0.00154 (-0.00314/-0.00102)	-0.00161(-0.00288/-0.00099)	-0.00214 (-0.00468/-0.00111)	< 0.001

*Abbreviations*: *Healthy* collective of young vascular healthy individuals (*n* = 23), *Kidney1* collective of patients with chronic kidney disease, fistula and intermediate extent of comorbidities (*n* = 39), *Kidney2* collective of patients with fistula on hemodialysis and high level of comorbidities (*n* = 46), *AI* Aortic index, *A2*_*non-fist*_ Area under the curve of the normalized pressure pulse curve between the systolic maximum and the end of systole measured at non-fistula arms, *m2*_*non-fist*_ mean slope of the normalized pressure pulse curve between the systolic maximum and the end of systole measured at non-fistula arms, *p* p-value for inter-collective comparison

No significant correlation was observed between AI and A2 or m2. However, a significant correlation was identified between A2 and m2 (r = 0.564, *p* < 0.001).

### Reactive hyperemia versus normal conditions

**Fig. 1:**
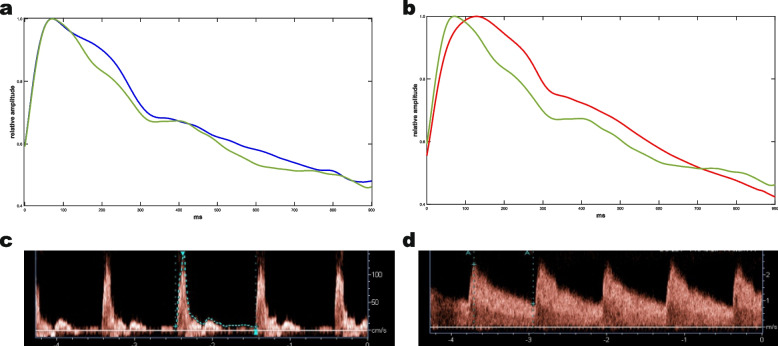
Exemplary measurements in a single patient. Pulse wave under reactive hyperemia versus physiological condition (**a**), fistula versus non-fistula arm (**b**) and duplex sonography (**c**: physiological condition, **d**: reactive hyperemia)

Eexemplary typical images for both, PWA and duplex sonography, are presented in Fig. 1.

The medians of ∆A2, ∆m2, ∆V_max_ and ∆V_diast_ (parameter differences in the hyperemic state as compared to the physiological state) differed significantly from zero in the mixed collective as well as in each single collective (Healthy and Kidney1). Also, the values differed significantly between the Healthy and kidney 1 collective (Table 3).

**Table 3:** Hyperemic versus physiological state

_**reac/phys**_	**Mixed Collective ***n* = 62		**Healthy ***n* = 23		**Kidney1** *n* = 39		**Comparison** (Healthy/Kidney1)
	**Median** (min/max)	*p*	**Median** (min/max)	*p*	**Median** (min/max)	*p*	*P*
**∆A2** [relative amplitude*s]	**0.00979** (-0.00986/0.03944)	< 0.001	**0.01877** (-0.00706/0.03705)	< 0.001	**0.00705** (-0.00986/0.03944)	< 0.001	< 0.001
**∆m2** [relative amplitude/s]	**0.00039** (-0.00033/0.00168)	< 0.001	**0.00052** (0.00025/0.00168)	< 0.001	**0.00029** (-0.00033/0.00080)	< 0.001	< 0.001
**∆T**_**max**_ [ms]	**21.6** (-33.3/125.5)	< 0.001	**23.5** (-33.3/115.7)	0.001	**19.6** (-23.5/125.5)	< 0.001	0.347
**∆V**_**max**_ [cm/s]	**74.2** ( -46.1/154.0)	< 0.001	**94.6** (36.2/154.0)	< 0.001	**63.4** (-46.1/122.4)	< 0.001	0.002
**∆V**_**diast**_ [cm/s]	**45.2** (16.60/98.5)	< 0.001	**58.9** (39.1/98.5)	< 0.001	**37.8** (16.6/93.9)	< 0.001	< 0.001

*Abbreviations*: *reac/phys* the hyperemic/physiological state, *A2/m2* AUC/mean slope of the normalized pressure pulse curve between the systolic maximum and the end of systole, *T*_*max*_ time point of systolic maximum, *V*_*max*_*/V*_*diast*_ maximum/diastolic flow velocity, *∆* indicating parameter inter-state difference (reac/phys), *p* p-value for inter-state testing, *P* p-value for inter-collective testing, *Healthy* collective of young vascular healthy individuals, *Kidney1* collective of patients with chronic kidney disease and fistula

**Fig. 2:**
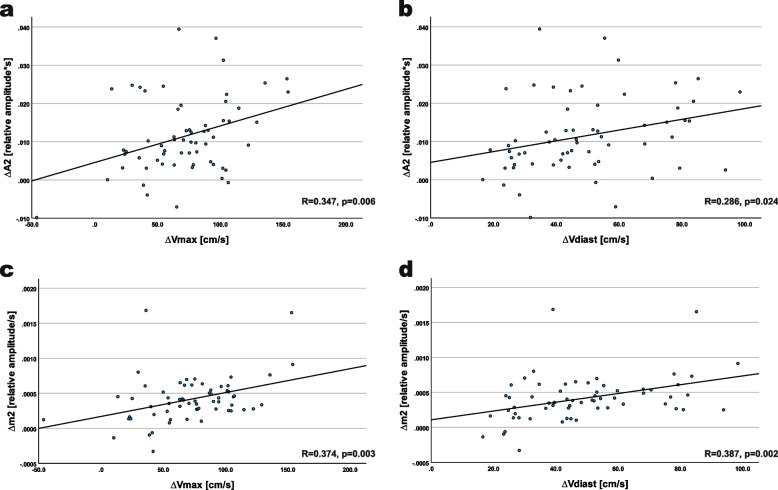
Relation of PWA and duplex parameter differences (hyperemic versus physiological state). Scatterplots of ∆A2 (panel **a** and **b**) and ∆m2 (panel **c** and **d**) in relation to ∆Vmax (panel **a** and **c**) and to ∆Vdiast (panel **b** and **d**); given significant correlation, regression lines are added (regression coefficient r and *p*-value in the bottom of each panel)

There was a significant correlation between ΔA2 and Δm2 and both ΔV_max_ (r = 0.347, *p* = 0.006 and r = 0.374, *p* = 0.003) and ΔV_diast_ (r = 0.286, *p* = 0.024 and r = 0.387, *p* = 0.002) and furthermore with each other (r = -0.531, *p* < 0.001). Additionally, there was a significant correlation between ΔV_max_ and ΔV_diast_ (r = 0.819, *p* < 0.001). Linear regression plots for ΔA2 and Δm2 versus ΔV_max_ and ΔV_diast_ are depicted in Fig. 2.

AI (measured at the radial artery in the physiological state, see methods and Protocol) correlated with ∆A2 (r = 0.445, *p* < 0.001), ∆m2 (r = 0.338, *p* = 0.007), ∆V_max_ (r = 0.460, *p* < 0.001) and ∆V_diast_ (r = 0.472, *p* < 0.001).

The evaluation of ROC analysis for endothelial dysfunction assessed with ΔV_max_ (cut- off 58.29 cm/sec, see method Sect. "Statistical analyses") revealed a significant result for Δm2 (AUC 0.706, *p* = 0.025, confidence interval 0.526–0.886), but neither for ΔA2 nor for AI. Nevertheless, ROC-Analysis using ΔV_diast_ as an indicator for endothelial dysfunction did not yield significant results.

### The impact of Fistula flow

The medians of ∆A2, ∆m2, ∆V_max_ and ∆V_diast_ (parameter differences of measurements at the fistula and the contralateral non-fistula arm) differed significantly from zero in the mixed collective of patients from the Kidney1 and Kidney2 collective as well as in both single collectives. No significant inter-collective differences were observed (Table 4).

**Table 4:** Fistula versus non-fistula measurements

_**fist/non-fist**_	**Mixed Cohort** *n* = 85		**Kidney1** *n* = 39		**Kidney2** *n* = 46		Comparison (Kidney1/Kidney2)
	**Median** (min/max)	*p*	**Median** (min/max)	*p*	**Median** (min/max)	*p*	*P*
**∆A2** [relative amplitude*s]	**0.00994** (-0.03811/0.03860)	< 0.001	**0.01115** (-0.00973/0.03860)	< 0.001	**0.00666** (-0.03811/0.03004)	< 0.001	0.310
**∆m2** [relative amplitude/s]	**0.00038** (-0.00052/0.00331)	< 0.001	**0.00031** (-0.00024/0.00174)	< 0.001	**0.00047** (-0.00052/0.00331)	< 0.001	0.161
**∆T**_**max**_ [ms]	**15.7** (-90.2/145.1)	< 0.001	**11.8** (-29.4/111.8)	< 0.001	**17.6** (-90.2/145.1)	0.002	0.860
**∆V**_**max**_ [cm/s]	**60.8** (-33.9205.8)	< 0.001	**61.9** (-6.8/179.3)	< 0.001	**58.8** (-33.9/205.8)	< 0.001	0.528
**∆V**_**diast**_ [cm/s]	**61.1** (17.1/131.7)	< 0.001	**67.0** (17.1/129.4)	< 0.001	**59.9** (18.0/131.7)	< 0.001	0.315

*Abbreviations*: *fist/non-fist* fistula/non-fistula, *A2/m2* AUC/mean slope of the normalized pressure pulse curve between the systolic maximum and the end of systole at the radial artery, *T*_*max*_ time point of systolic maximum, *V*_*max*_/V_*diast*_ maximum/diastolic flow velocity at the brachial artery, *∆* indicating parameter difference between measurement at the fist and non-fist arm, *p* p-value for inter-state testing, *P* p-value for inter-collective testing, *Kidney1* collective of patients with chronic kidney disease, fistula and intermediate extent of comorbidities, *Kidney2* collective of patients with fistula on hemodialysis and high level of comorbidities

**Fig. 3:**
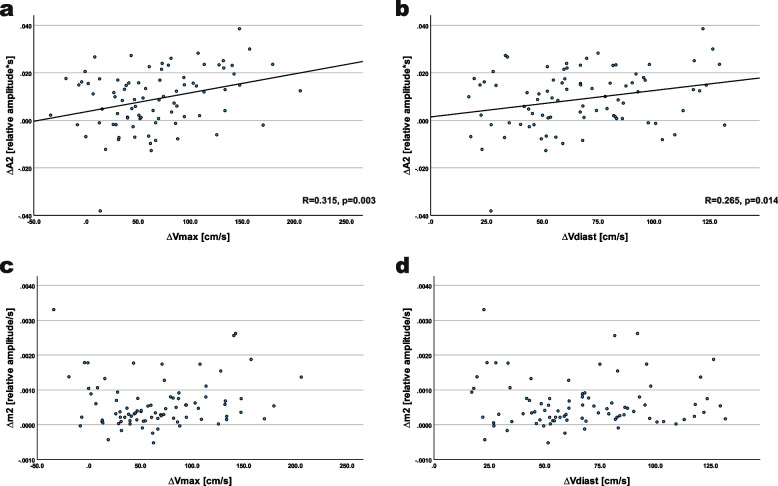
Relation of PWA and duplex parameter differences between the fistula and non-fistula arm. Scatterplots of ∆A2 (panel **a** and **b**) and ∆m2 (panel **c** and **d**) in relation to ∆Vmax (panel **a** and **c**) and to ∆Vdiast (panel **b** and **d**); in case of significant correlation, regression lines are added and the regression coefficient r and p-value are presented in the bottom of the respective panel

A significant correlation was observed between ΔA2 and both ΔV_max_ (r = 0.315, *p* = 0.003) and ΔV_diast_ (r = 0.265, *p* = 0.014), as well as with Δm2 (r = 0.509, *p* < 0.001). There was no significant correlation between Δm2 and ΔV_max_ or ΔV_diast_. Figure 3 depicts scatter plots for ΔA2 and Δm2 versus ΔV_max_ and ΔV_diast_, with regression lines indicating a significant correlation.

### Comparative analysis of the impact of hyperemic and fistula flow in the Kidney1 collective

Alterations in the pulse waveform under reactive hyperemia resembled those at the fistula arm (Fig. 1).

As with the measurements taken at the radial artery (see Results), the parameter differences (ΔA2 and Δm2) at the brachial artery of the fistula and the contralateral non-fistula arm were found to differ significantly from zero in the Kidney1 collective (Table 5). In comparing the hyperemic and fistula states, significant differences were observed for Δm2 and ΔV_diast_, but not for ΔA2 and ΔV_max_ (Table 5).

**Table 5:** Comparison of the three different hemodynamic states in the Kidney1 collective

*N* = 39	reac/phys		fist/phys		fist/reac	
	**Median** (min/max)	*p*	**Median** (min/max)	*p*	**Median** (min/max)	*p*
**∆A2**_**[**mmHG*s]_	**0.00705** (-0.00986/0.03944)	< 0.001	**0.00395** (-0.01526/0.03444)	< 0.001	**-0.00306** (-0.02149/0.01610)	0.100
**∆m2**_**[**mmHG/s]_	**0.00029** (-0.00033/0.00080)	< 0.001	**0.00029** (-0.00023/0.00114)	< 0.001	**0.00020** (-0.00062/0.00089)	0.005
**∆T**_**max**_ [ms]	**19.6** (-23.5/125.5)	< 0.001	**11.7** (-41.2/102.0)	0.003	**-7.8** (-131.4/98.0)	0.622
**∆V**_**max**_ [$\frac{cm}{s}$]	**63.4** (-46.1/122.40)	< 0.001	**61.9** (-6.80/179.30)	< 0.001	**12.2** (-94.0/80.3)	0.298
**∆V**_**diast**_ [$\frac{cm}{s}$]	**37.8** (16.6/93.9)	< 0.001	**67.0** (17.10/129.40)	< 0.001	**27.4** (-50.0/96.5)	< 0.001

*Abbreviations*: *Kidney1* collective of patients with chronic kidney disease and fistula, *A2/m2* AUC/mean slope of the normalized pressure pulse curve between the systolic maximum and the end of systole at the brachial artery, *T*_*max*_ time point of systolic maximum, *V*_*max*_/V_*diast*_ maximum/diastolic flow velocity at the brachial artery, *phys* the physiological state, *reac* the hyperemic state, *fist* the state close to an arteriovenous fistula, *∆* parameter-differences for the 3 state combinations reac/phys, reac/fist and fist/phys as indicated in the respective column heading, *p* p-value

### Time point of T_max_

A comparison of the state of reactive hyperemia versus physiological conditions, as well as of the fistula measurement versus the non-fistula measurement, revealed that T_max_ was greater in the respective high flow state in all collectives (Tables 3 and 4). In the kidney 1 collective, this was observed for both the radial and brachial measuring sides (Tables 4 and 5). The comparison of the state in proximity to a fistula and under reactive hyperemia yielded no significant differences (Table 5).

## Discussion

The presented findings document the impact of high flow on the pulse waveform using the SpygmoCor™ device. Changes in the AUC and mean slope in the second section of the pressure pulse waves (A2 and m2) could be demonstrated, which differed significantly in the hyperemic and physiological state as well as between fistula and contralateral non-fistula arms. These flow-induced changes in the newly established pulse wave parameters were demonstrated in three different collectives that differed in age, comorbidities and AI as a classical pulse wave parameter indicating vascular stiffness. Furthermore, the novel PW parameter correlated with the referential duplex parameters in both high-flow states except for m2 in the fistula state.

The extent of post-ischemic hyperemic flow depends on endothelial function and the increment in peak and end-diastolic flow velocity assessed by duplex sonography is an established tool to characterize the post-ischemic reaction of the endothelium [22, 23]. The results presented here suggest, that also PWA in reactive hyperemia may be useful to evaluate endothelial function. As indicated by clinical parameters and higher AI, the older and more morbid Kidney1 collective most likely has a reduced endothelial function as compared to the Healthy collective. Consistent with this, the hyperemia induced alterations in pulse wave morphology and in duplex flow velocities were greater in the latter collective. Moreover, we demonstrated that ∆m2 potentially is suitable to detect a duplex-defined cut-off value indicative of endothelial dysfunction.

AI is usually considered to be a parameter influenced by vascular stiffness. Nevertheless, there is some evidence, that AI is related to endothelial function because endothelial function influences arteriolar tone and hence wave reflection [24]. Although AI was not proven to detect endothelial dysfunction in the ROC analysis, our findings support this theory: AI differed between the Healthy and Kidney1 collectives and moreover, there was significant correlation between AI and all assessed parameter differences between the hyperemic and physiological states (but not with A2 and m2 in the physiological state). From a clinical perspective, it is important to note that increased vascular stiffness and endothelial dysfunction often occur simultaneously [25] and share pathogenetic factors [26]. Further studies are needed to determine whether AI is more influenced by vascular stiffness or by endothelial dysfunction and whether AI, ΔA2 and Δm2 are of additive clinical value.

A small study examined the impact of inhaled albuterol on endothelial function and associated alterations in pulse waveform and like the presented study found promising results [15]. Taken together, the approach propounded here offers the possibility to specifically evaluate endothelial function and established parameters of PWA with a single device, which is well introduced into scientific and clinical practice without the necessity of pharmacological induced modification of endothelial function which may be contraindicated especially in high-risk patients. By analyzing digitized PWs, we were not limited to the classical parameters readily provided by the device, but were able to establish novel parameters for the analysis of peripheral pulse waves that may be more suitable in the context of local reactive hyperemia than those readily provided by the SphygmoCor© device: For example, AI is a measure of central augmentation due to pulse wave reflection in distributed parts of the entire peripheral vasculature.

The analysis of the Kidney1 collective offers the opportunity to compare three peripheral hemodynamic states, namely the physiological state and two high flow states, i.e. the hyperemic state and the fistula-induced state. Most interesting is the comparison of the two high flow states. As suggested by the optical similarity (Fig. 1), no significant parameter difference between the hyperemic and fistula states could be demonstrated for ΔA2, ΔT_max_ and ΔV_max_, albeit for Δm2 and ΔV_diast_. Although the results are inconclusive from a clinical point of view, they may stimulate studies to test the hypothesis that PWA can simulate fistula flow under conditions of reactive hyperemia. Postoperative fistula maturation depends, among other factors, on the ability of the conducting artery to increase flow [27] and Malovrh demonstrated, that preoperative post-ischemic increase of upper arm flow in duplex sonographic evaluation can predict postoperative primary patency rates [28]. Our current findings suggest, that pre-operative post-ischemic PWA may serve as a more convenient alternative.

Generally speaking, our data highlight the effect of exceptionally low peripheral resistance and high flow on the pulse waveform. In terms of a more comprehensive clinical application, it would be interesting to design studies analyzing more subtle hemodynamic variations, for example using big data from wearables or monitoring devices in intensive care.

The question arises as to what mechanisms cause the observed changes in pulse wave contours under high flow conditions. Since both high-flow scenarios are accompanied by an arterial dilation, a likely explanation for the observed flow related changes in pulse waveform is an increase in brachial artery windkessel function. Also pulse wave propagation velocity must be considerd: Naka et al. demonstrated a reduction in pulse wave propagation velocity under reactive hyperemia [29] and we have shown that the pulse wave propagation velocity is lower in the high-flow fistula state than in the physiological state [19]. This may be an explanation for the delay in T_max_ now observed in the high-flow states. Moreover, a reduced pulse wave propagation velocity goes along with a prolonged time lag in pulse wave reflection. Consequently, the overlap of the antegrade and reflected waves occurs later, which partially may explain the observed alterations in the pulse waves between T_max_ and end of systole. Pulse waveform changes due to reactive hyperemia or pharmacologic peripheral vasodilation have also been interpreted as a consequence of reduced amplitudes of reflected pulse waves [5, 24]. It is beyond the scope of our analysis to evaluate pulse wave reflection in depth. In this regard it would be interesting to measure impedance by simultaneous continuous recording of flow and pressure in future studies, as Collard et al. recently did in the renal artery [30].

### Limitations

Our study has several limitations. First, it was an explorative analysis without predefinition of the novel parameters. Second, an extrapolation to other mixed cohorts or high risk collectives is not possible without validating larger clinical trials including non-renal patients. Third, measuring PWA under reactive hyperemia with the SpygmoCor™-device was challenging and could be cumbersome in clinical practice. Fourth, we used duplex sonography to referentially evaluate endothelial function under reactive hyperemia which has been proven to be of predictive value in the Framingham collective and was useful to characterize the high flow condition [11]. Nevertheless, sonographic measurement of flow-mediated dilatation is more widely used to access endothelial function. Fifth, as discussed in detail above, there may be an interaction between novel and established PWA parameters, probably depending on the vascular stiffness of the studied collective [25, 31]. Future analysis will need to determine, if the novel parameters can predict clinical outcomes, if there are even more appropriate PW parameters to be computed from the digitized PWs and if machine learning is a booster for assessment of pulse wave under the conditions of high flow.

### Conclusion

There are characteristic alterations in the second section of pulse waves under the condition of reactive hyperemia as well as in the vicinity of an arteriovenous fistula. These were characterized by newly computed parameters, could be related to an increase in flow velocity and be demonstrated in a healthy collective as well as in two collectives of patients with renal disease and a different level of comorbidities.

## Data Availability

The datasets used and analysed during the current study as well as the applied matlab™ code to assess digitised pulse waves are available from the corresponding author on reasonable request.

## Abbreviations

PWA: Pulse wave analysis
AVF: Arterio-venous fistula
m2: Mean slope between the systolic maximum and the end of systole
A2: Area under the curve between the systolic maximum and the end of systole
V_max_: Systolic peak flow velocity
V_diast_: Diastolic flow velocity
